# Spotty liver disease adversely affect the gut microbiota of layers hen

**DOI:** 10.3389/fvets.2022.1039774

**Published:** 2022-10-28

**Authors:** Thi Thu Hao Van, Lisa F. M. Lee Nen That, Rachelle Perera, Arif Anwar, Timothy B. Wilson, Peter C. Scott, Dragana Stanley, Robert J. Moore

**Affiliations:** ^1^School of Science, RMIT University, Bundoora, VIC, Australia; ^2^Scolexia Pty Ltd., Moonee Ponds, VIC, Australia; ^3^Institute for Future Farming Systems, Central Queensland University, Rockhampton, QLD, Australia

**Keywords:** spotty liver disease, microbiota, layers, chickens, *Campylobacter*, gut health

## Abstract

Spotty Liver Disease (SLD) is a serious infectious disease which occurs mainly in laying chickens in free range production systems. SLD outbreaks can increase mortality and decrease egg production of chickens, adversely impact welfare and cause economic hardship for poultry producers. The bacterium *Campylobacter hepaticus* is the primary cause of the disease. This study aimed to identify the effects of *C. hepaticus* on chicken gut microbiota and gut structure. Three *C. hepaticus* strains (HV10^T^, NSW44L and QLD19L), isolated from different states of Australia, were used in the study. Chickens at 26-weeks post-hatch were orally dosed with one of the *C. hepaticus* strains (challenged groups) or Brucella broth (unchallenged or control group). Six days after the challenge, birds were necropsied to assess liver damage, and caecal content and tissue samples were collected for histology, microbiology, and 16S rRNA gene amplicon sequencing to characterize the composition of the bacterial microbiota. Strain *C. hepaticus* NSW44L produced significantly more disease compared to the other *C. hepaticus* strains and this coincided with more adverse changes observed in the caecal microbiota of the birds challenged with this strain compared to the control group. Microbial diversity determined by Shannon and Simpson alpha diversity indices was lower in the NSW44L challenged groups compared to the control group (*p* = 0.009 and 0.0233 respectively, at genus level). Short-chain fatty acids (SCFAs) producing bacteria *Faecalibacterium, Bifidobacterium* and *Megamonas* were significantly reduced in the challenged groups compared to the unchallenged control group. Although SLD-induction affected the gut microbiota of chickens, their small intestine morphology was not noticeably affected as there were no significant differences in the villus height or ratio of villus height and crypt depth. As gut health plays a pivotal role in the overall health and productivity of chickens, approaches to improve the gut health of the birds during SLD outbreaks such as through diet and keeping the causes of stress to a minimum, may represent significant ways to alleviate the impact of SLD.

## Introduction

Spotty Liver Disease (SLD), causes small whitish-gray miliary spots on the surface of the liver, and is a significant disease in poultry. It causes significant loss to the poultry industry as it can cause up to 10% flock mortalities and 25% reduction in egg production in flocks experiencing a disease outbreak (1, 2). Even though SLD was noted decades earlier, its specific etiology was recognized only in 2015, when a novel *Campylobacter* was isolated from SLD-affected chickens (3). Following this discovery, Van et al. (4) isolated, characterized, and formally named a new bacterium isolated from SLD affected birds in Australia (4). This specific bacterium was named *Campylobacter hepaticus*. *C. hepaticus* was confirmed as the cause of SLD after experiments showing that the Koch's Postulates were fulfilled (5). Unlike *C. jejuni* and *C. coli*, which are mostly commensal in chickens but pathogenic in human, *C. hepaticus* causes spotty liver disease in chickens but pathogenicity to humans has not been reported.

*C. hepaticus* is a Gram-negative, S-shaped bacterium which grows under microaerobic conditions. It has singular bipolar flagella and ranges in length between 1 and 1.2 um and width of 0.3–0.4 um (4). Compared to other *Campylobacter* species, *C. hepaticus* has a small genome size of ~1.5 Mb and a lower G+C content, between 27.9 and 28.5%, depending on the strain (6). *C. hepaticus* colonizes the small intestine of infected chickens, increasing in population from duodenum to ileum, and is at the highest levels within the caeca. They were also readily detected in cloacal swabs, indicating it is likely transmitted *via* a fecal-oral infection (7). The bacterium can be cultured from the liver and bile of infected birds. Isolation of *C. hepaticus* from complex primary microbial sources has proven to be difficult due to the absence of a fully selective media and growth of faster growing contaminating microorganisms (4, 8). Chickens entering the peak lay period, between 22 and 27 weeks, were found to be highly susceptible to SLD outbreaks. They can be infected with the responsible bacterium as young as 12 weeks old and up to 8 weeks before clinical SLD is manifested (8). *C. hepaticus* has shown the potential to survive for long periods in the farm environment by entering viable but non-culturable (VBNC) states (9).

The gastrointestinal tract (GI) of chickens contains diverse and complex microbiota which contribute to digestion and absorption of nutrients, immune system development, and pathogen exclusion (10). The functionality and the health of chicken gut is dependent on the surrounding environment, feed, and the GI microbiota. Chicken GI microbiota plays a pivotal role in the maintenance of intestinal health and can form a protective barrier by attaching to the GI tissues to reduce the opportunity for colonization of pathogenic bacteria (11). A healthy structure and function of chicken intestinal microbiota is crucial for the positive production performance of poultry (12, 13).

This study aimed to investigate the gut health of chickens by determining whether the SLD pathogenesis resulted in changes in the caecal microbiota by comparing the microbiota of an unchallenged group with *C. hepaticus* infected groups which were challenged with three different *C. hepaticus* strains. The effect of SLD on gut morpho-histology was also studied. The understanding of changes in gut microbiota and structure during SLD will help to direct the development of intervention strategies, such as feed modifications or probiotic application, to improve the gut health of the chickens.

## Materials and methods

### Bacterial strains and growth conditions

*C. hepaticus* HV10^T^, *C. hepaticus* NSW44L and *C. hepaticus* QLD19L were grown on Brucella agar (Becton Dickinson) with 5% horse blood (HBA) and incubated at 37 °C in microaerobic conditions using CampyGen gas packs (Oxoid) for 3 days (6). To prepare the inoculum for the chicken challenge, the culture was grown in Brucella broth supplemented with l-cysteine (0.4 mM), and l-glutamine (4 mM) and sodium pyruvate (10 mM) (modified Brucella broth) in 75 cm^2^ tissue culture flasks at 37°C for 48 h in microaerophilic conditions and used directly for the challenge, as previously described (14).

### Chicken challenge experiments

A *C. hepaticus* challenge trial of 26-week old Hy-Line layer hens was carried out to investigate the changes in gut microbiota and gut structure of the *C. hepaticus* unchallenged group (control group) compared to challenged groups, where birds were challenged with HV10^T^, NSW44L or QLD19L strains. Birds from each group were housed in 4 cages, 3 birds per cage. Birds were sourced from a farm that had not observed any SLD in their flocks for several years and birds were confirmed as *C. hepaticus*-negative by specific PCR of fecal material collected by cloacal swabbing, using the previously published protocol (5). The animal experiment was approved by the Wildlife and Small Institutions Animal Ethics Committee of the Victorian Department of Economic Development, Jobs, Transport and Resources (approval number 14.16). The challenges were carried out as described in (14). In brief, the control chickens were given 1 mL of modified Brucella broth, the challenged groups were dosed with 1 x 10^9^ CFU of the relevant *C. hepaticus* strain in 1 mL of modified Brucella broth. The birds were fed *ad libitum* with a standard, antibiotic-free layer diet. There were 12 birds each in the negative control and *C. hepaticus* HV10^T^ groups, and 8 birds for the *C. hepaticus* NSW44L and *C. hepaticus* QLD19L groups.

At 6 days post infection, the birds were euthanized by cervical dislocation and the livers were examined for lesions. Liver lesion score was measured by the number of spots on a liver: a score of 0 indicated no spots; 1 indicated 1–5 spots; 2 indicated 6–20 spots; 3 indicated 21–100 spots; 4 indicated 101–1,000 spots; and 5 indicated more than 1,000 spots. Caecal content samples were collected and stored at −20°C until further analysis. Bile samples were taken aseptically from the gall bladder, kept on ice and processed within 3 h. Isolation from bile samples was performed as previously described (5), briefly, 20 μL of bile was spread directly onto horse blood agar plates and incubated at 37°C in microaerobic conditions for 3 days and confirmed by MALDI-TOF. Tissue samples were taken from the distal end of the jejunum of each bird and preserved in 10% neutral phosphate-buffered formalin for histology.

### Histology

The tissue samples were embedded, sectioned, and hematoxylin and eosin stained by Gribbles Pathology (Australia). The control group, HV10^T^, and NSW44L groups were investigated. The mounted tissue sections were examined and scored for villus height and crypt depth. Ten villus/crypt measurements were made for each bird. The histology data were analyzed using one-way ANOVA in Minitab 21. The Dunnett test was used to compare every mean to a control mean.

### 16S rRNA gene amplicon sequencing and data analysis

DNA from the caecal samples was extracted using a Bioline Isolate Fecal DNA kit (Meridian, cat.no#BIO-52082). Primers were selected to amplify the V3–V4 region of 16S rRNA genes: forward ACTCCTACGGGAGGCAGCAG and reverse GGACTACHVGGGTWTCTAAT, and also contained barcodes, spacer and Illumina sequencing linker sequences as detailed previously (15). PCR was carried out in a final volume of 20 μL using Q5 High-Fidelity 2× Master Mix (New England Biolabs), primers at a final concentration of 250 nM each and 1 μL of template DNA. An Eppendorf Mastercycler Pro PCR instrument was used for amplification with cycling conditions of; 98°C for 1 min, 35 cycles of 98°C for 10 s; 49°C for 30 s and 72 °C for 30 s, and final extension at 72 °C for 10 min. Negative controls (water) were added in the sample preparation process and no amplicons were produced from the negative controls. Sequencing was performed on an Illumina MiSeq platform using 2 x 300 bp paired-end sequencing.

Sequence data were trimmed with Trimmomatic (v 0.39) and then fastq files were analyzed using DADA2 in QIIME2 v2020.6 (16) to denoise and produce Amplicon Sequence Variants (ASVs). Quality filtering was applied with the default option. ASVs were clustered at 99% identity using the VSEARCH plugin (17). Taxonomy was assigned using the SILVA database (v138). A total of 940,924 high-quality paired-end reads were obtained, with an average of 23,523 reads per sample, with a minimum of 10,023 and a maximum of 36,955. Obtaining feature table was further filtered (features that were present in only a single sample and samples with a total frequency <1,000). A total of 9,624 OTUs were found. The downstream statistical microbial data analyses and visualizations were done using MicrobiomeAnalyst (18), where features with mean values of <4 were filtered. The community profiling was based on the R Phyloseq and Vegan packages. Principal coordinates analysis (PCoA) (Jaccard Index and PERMANOVA) was used to visualize the clustering of samples based on their genus-level compositional profiles. Associations of specific bacteria within each group were identified using the linear discriminant analysis effect size and the multivariate analysis implemented in the MicrobiomeAnalyst software (18). The sequence data used for analysis is available in NCBI under BioProject accession number PRJNA877767.

## Results

### *C. hepaticus* NSW44L produced significant more SLD clinical disease compared to other *C. hepaticus* strains

At 6-days post challenge, the birds were euthanized and the livers were accessed for typical SLD lesion. As expected, all unchallenged birds had normal healthy livers with no SLD lesions. All birds from the NSW44L challenged group had typical SLD liver lesions, with liver scores of 4 and 5 for all birds (mean liver score of 4.375), whereas 2 birds in the HV10^T^ groups didn't have lesions on the livers and the average liver score for the group was 2.57, all birds from QLD19L had SLD liver lesions, but both HV10^T^ and QLD19L challenged groups had significantly lower liver score than the NSW44L group (*p* < 0.05, Table 1). *C. hepaticus* was isolated from all challenged birds, except one from the QLD19L challenged group.

**Table 1:** Live lesion scores of birds challenged with different *C. hepaticus* strains.

**Group**	**Mean liver** **lesion** score^*^	**StDev**	**95% CI**
HV10^T^	2.583^a^	1.832	(1.771, 3.396)
NSW44L	4.375	0.518	(3.380, 5.370)
QLD19L	3.375^a^	1.065	(2.380, 4.370)

* Means not labeled with the letter

a are significantly different from the mean score of HV10 group (p < 0.05).

### SLD had negative effects on the gut microbiota of the *C. hepaticus* NSW44L challenged group

**Figure 1:**
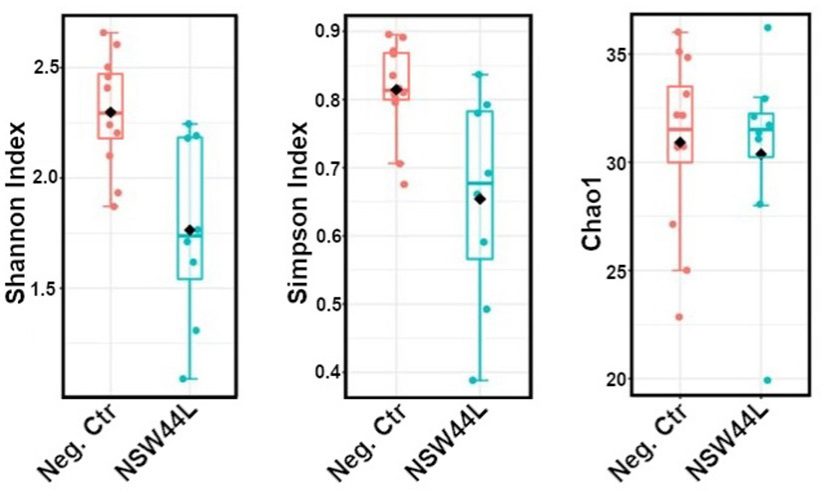
Alpha-diversity plots showed the *C. hepaticus* NSW44L challenged group has significantly lower Shannon and Simpson alpha diversity [*p* = 0.009 and 0.0233, respectively, **(left and middle panels)**] compared to the control group. No significant difference was found in the Chao1 richness index **(right panel)**.

The *C. hepaticus* NSW44L challenged group had significantly lower Shannon and Simpson alpha diversity values (*p* = 0.009 and 0.0233, respectively, at genus level) compared to the unchallenged control group. No significant difference was found in the Chao1 richness index between the control and NSW44 challenged groups (Figure 1).

**Figure 2:**
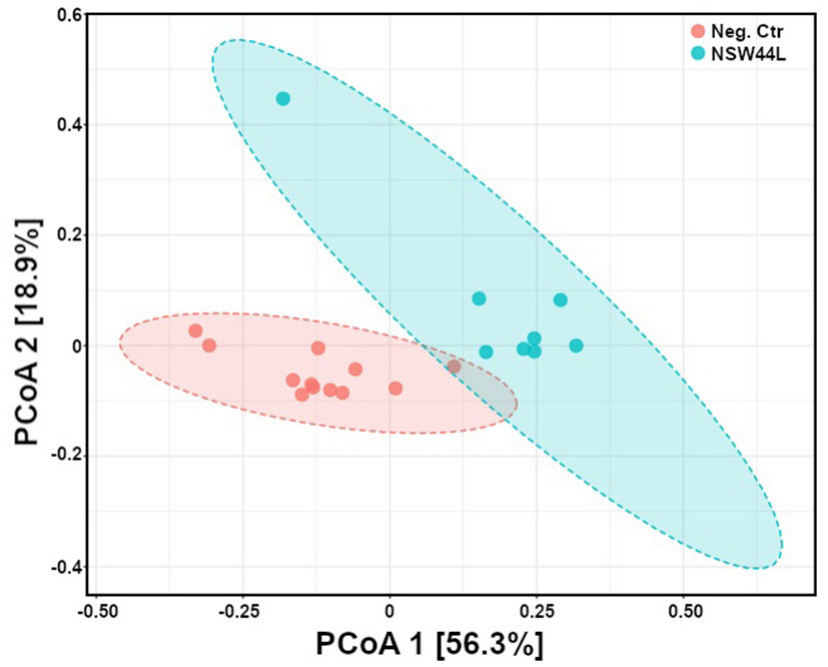
Principal coordinates analysis (PCoA) of gut microbiota composition of *C. hepaticus* challenged groups compared with non-challenged birds (at phylum level). The group challenged with the NSW44L strain was clustered away from the control group.

Principal Coordinate Analysis (PCoA) was used to explore and visualize similarities/dissimilarities in the overall microbiota compositions of the *C. hepaticus* NSW44L challenged group and the unchallenged control group. Gut microbiota compositions of the challenged birds were distinct from the non-challenged group (*p* < 0.001) (Figure 2).

**Figure 3:**
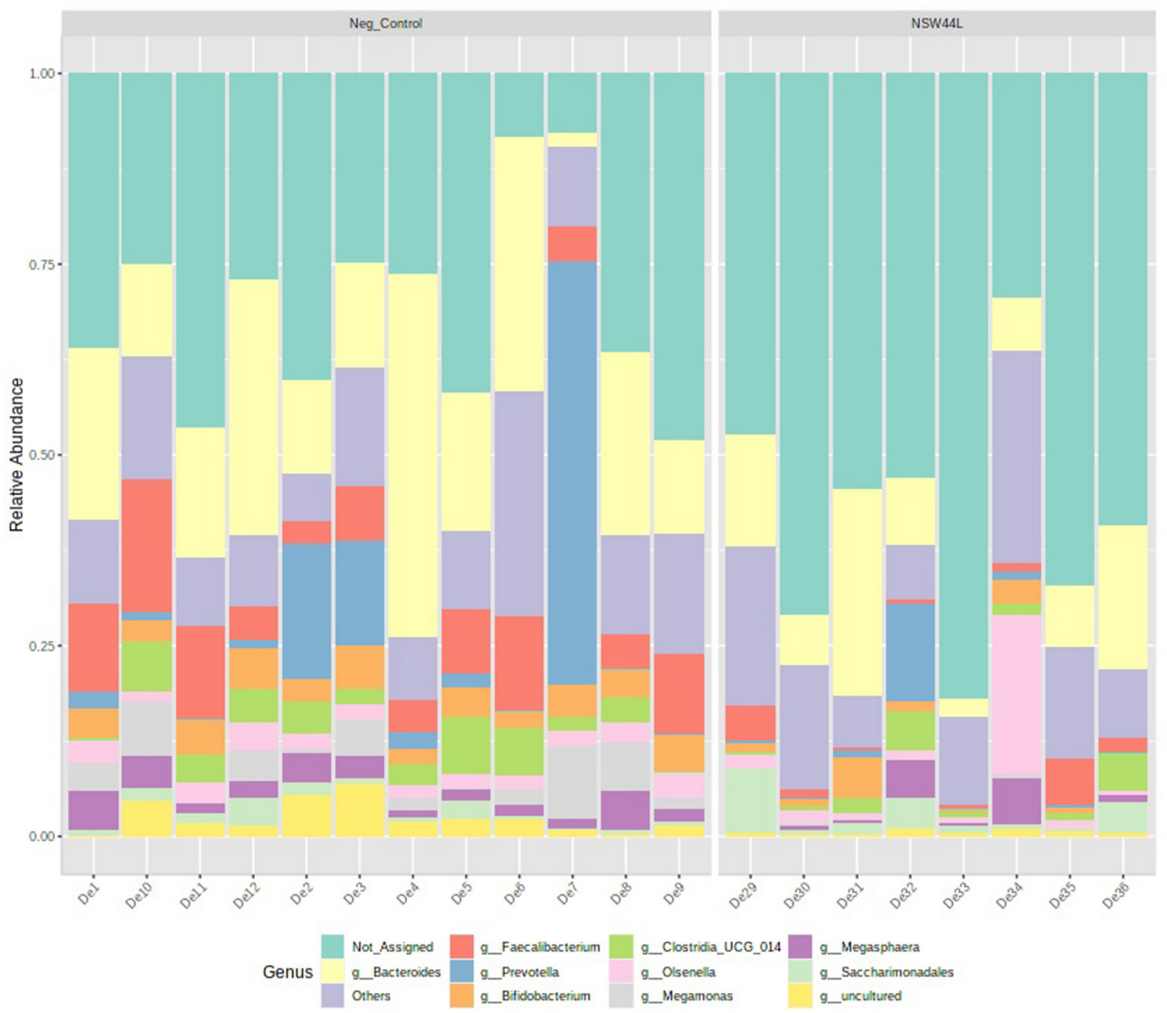
Relative abundance of top 10 genera detected in caecal content of unchallenged and *C. hepaticus* NSW44L challenged birds.

The relative abundance of the top 10 genera is visualized in a stacked bar chart and it can be seen that members of the *Faecalibacterium, Bifidobacterium* and *Megamonas* genera have reduced relative abundance in the *C. hepaticus* NSW44L challenged group compared to the unchallenged control group (presented in red, orange and purple respectively in Figure 3).

**Figure 4:**
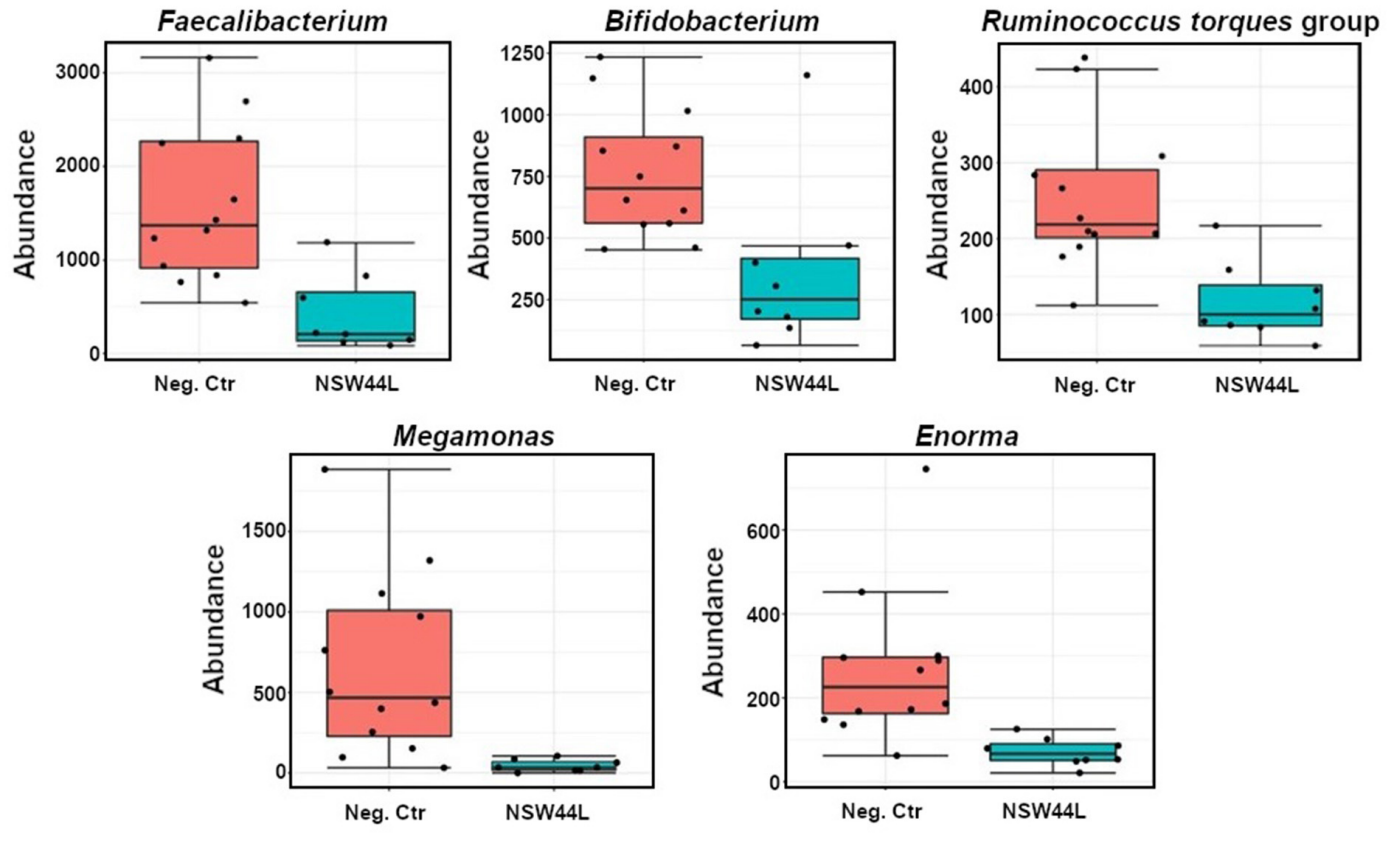
Classical univariate statistical comparisons showed significant reductions of *Faecalibacterium, Bifidobacterium, Ruminococcus torques* group*, Megamonas* and *Enorma* genera in the NSW44L challenged group.

Univariate analysis showed significant reductions in the relative abundances of *Faecalibacterium, Bifidobacterium, Ruminococcus torques, Megamonas* and *Enorma* genera in the challenged group compared to the unchallenged group (*p* < 0.05) (Figure 4).

### SLD also adversely affected the microbiota of birds challenged with different strains of *C. hepaticus*

To determine whether the changes in microbiota composition seen following infection with *C. hepaticus* NSW44L were unique to that particular treatment group or indicated a more general response to the challenge, two other pathogenic strains of *C. hepaticus*, QLD19L and the type strain HV10^T^, were used to infect layer birds. There was no significant difference in the bacteria diversity (Shannon and Simpson indexes) and Chao1 richness index among the control, HV10^T^ and QLD19L groups (data not shown).

**Figure 5:**
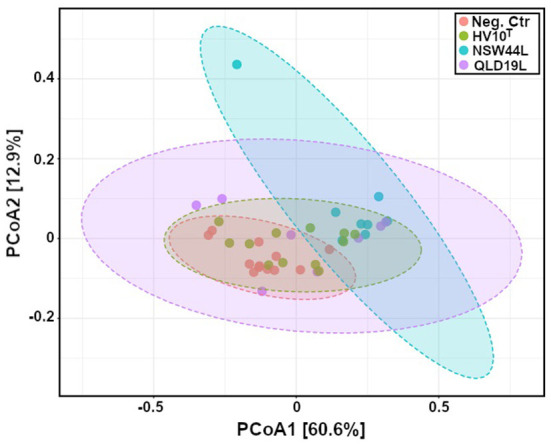
Principal coordinates analysis (PCoA) of gut microbiota composition of *C. hepaticus* challenged groups compared with non-challenged birds (p < 0.001). The group challenged with the NSW44L strain was clustered away from the control group, but the separation was not obvious for the other two challenged groups.

Principal Coordinates Analysis (PCoA) using a Jaccard distance matrix index showed gut microbiota composition of challenged NSW44 was distinct from the non-challenged group, but the separation was not apparent for the other two challenged groups. This result is consistent with disease severity as this group had significantly more liver lesions than the other groups (Figure 5).

**Figure 6:**
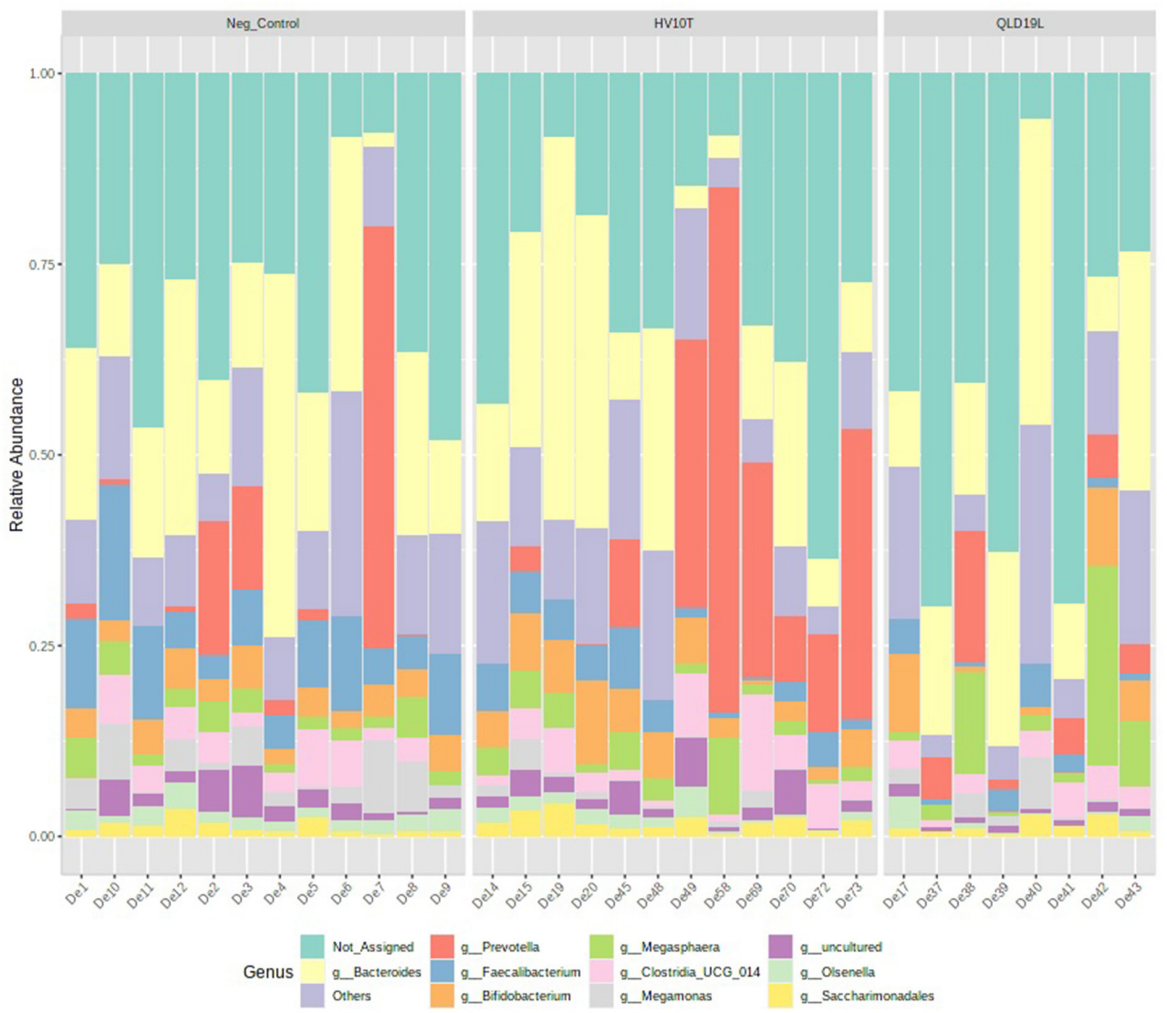
Relative abundance of top 10 genera detected in caecal content of unchallenged and *C. hepaticus* HV10^T^ and QLD19L challenged birds.

The relative abundance of the top 10 genera presented in Figure 6 showed that members of the *Faecalibacterium* and *Megamonas* genera had reduced relative abundance in the *C. hepaticus* HV10^T^ and QLD19L challenged groups compared to the unchallenged control group (presented in blue and purple respectively in Figure 6).

**Figure 7:**
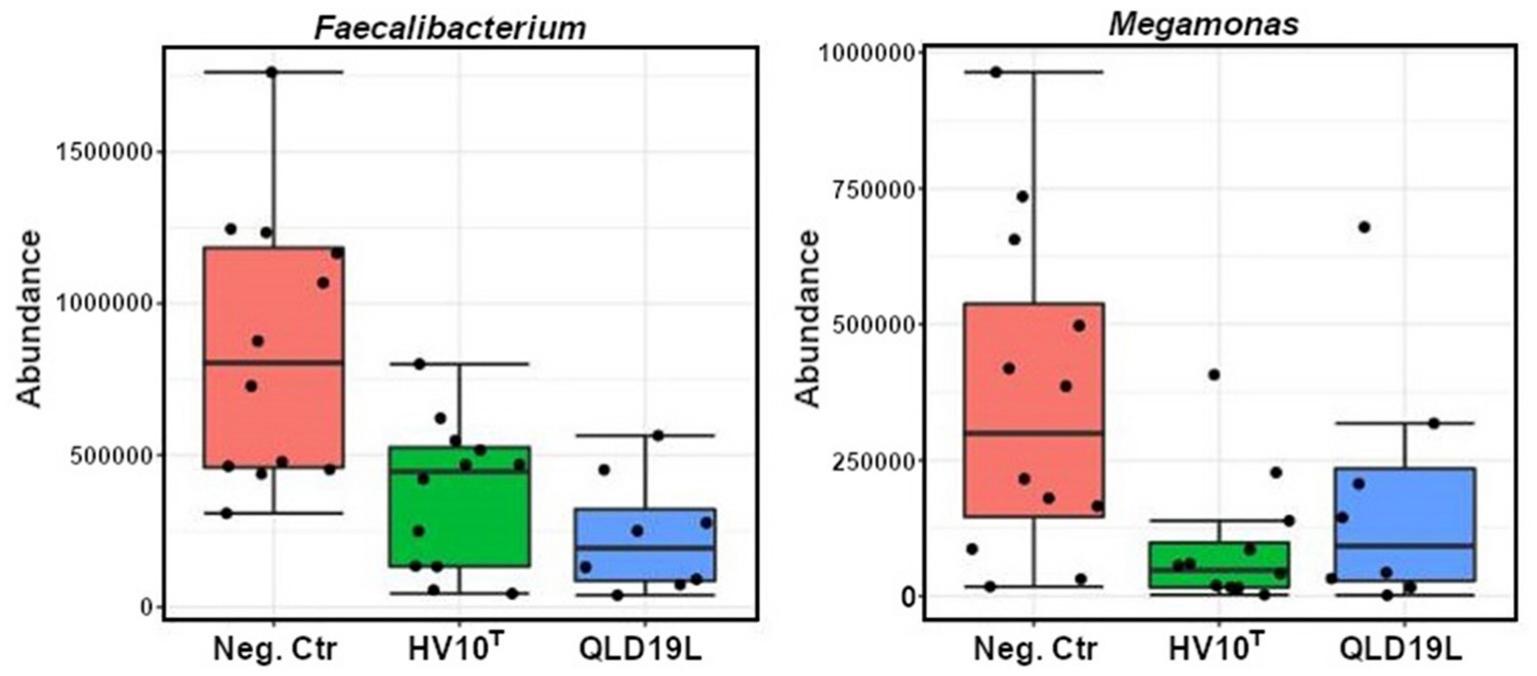
Classical Univariate Statistical Comparisons showed significant reductions of *Faecalibacterium* and *Megamonas* in the HV10T and QLD19L groups (*p* < 0.05).

Furthermore, the univariate statistical comparisons showed significantly reduced relative abundances of *Faecalibacterium*, and *Megamonas* in all challenged groups compared to the unchallenged control group (*p* < 0.05) (Figure 7).

### Gut histology

**Figure 8:**
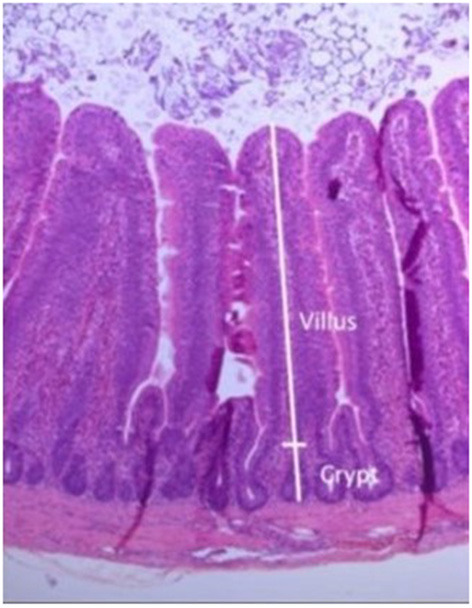
Gut section stained with hematoxylin and eosin, showing measurements taken of villus and crypt.

A typical gut histological section used to measure the villus height and crypt depth is shown in Figure 8.

The group mean measurements of villus heights and villus/crypt height ratios are shown in Table 2. No statistically significant differences between groups were observed for either villus height or villus/crypt ratios.

**Table 2:** Villus height and villus/crypt ratios.

**Group**	**Mean villus** **height**	**StDev of** **villus** **height**	**Villus** **height** **95% CI**	**Mean** **villus/crypt** **ratio**	**Villus/crypt** **ratio StDev**	**Villus/crypt** **ratio 95%** **CI**
Control	483.5	39.3	(428.4, 538.6)	6.844	0.834	(5.030, 8.658)
HV10^T^	570.0	48.0	(515.0, 625.1)	6.065	1.944	(4.251, 7.880)
NSW44L	565.7	57.1	(510.6, 620.8)	5.886	1.801	(4.072, 7.701)

## Discussions

*C. hepaticus* strains isolated from different states in Australia, including HV10^T^ (Victoria), NSW44L (New South Wales) and QLD19L (Queensland) were used to challenge birds at peak lay to examine the effect of SLD on gut health. The NSW44L strain produced significantly more liver lesions than the other two strains (*p* < 0.05). The genetic differences between different strains may play a role in disease pathogenesis, although genome analysis showed that the HV10^T^ and NSW44L strains belong to the same lineage, whereas QLD19L was in another lineage (6).

As gut microbiota has important functions of food digestion and production of essential nutrients, regulation of immune, metabolic and nervous systems, diverse gut bacteria are necessary to support these functions (11). The adverse effects of *C. hepaticus* on alpha diversity were observed in *C. hepaticus* NSW44L challenged group, as the Shannon and Simpson alpha diversities were significantly reduced in the challenged groups compared to the control group (*p* < 0.05). Beta diversity analysis indicated a significant difference (*p* < 0.001) in community structure between the control and NSW44_ challenged groups with clear separation of the clustered samples of each group. There was considerable overlap in the coordination of the samples in beta diversity between the unchallenged, HV10^T^ and QLD19L groups, indicating little systematic difference in the overall compositions of these caecal microbiotas. This apparent difference in the overall structure of the microbiota of the NSW44L infected group compared to the other two infection groups is consistent with the degree of pathogenicity of the *C. hepaticus* strains, as NSW44L strains produced significantly more disease than the other two strains.

When the microbiota communities of the challenged and unchallenged birds were investigated in more detail, it was observed that *Faecalibacterium* and *Megamonas* genera were reduced in the challenged groups compared to the non-challenged group. *Faecalibacterium prausnitzii* is the most well-characterized species of the *Faecalibacterium* genus, and other species have recently been identified and reported, including *F. moorei* (19), *F. butyricigenerans, F. hominis, F. longum* (20), *F. duncaniae, F. gallinarum*, and *F. hattorii* (21). Most *Faecalibacterium* bacteria are butyric acid producers, therefore they can exhibit anti-inflammatory effects and are potential probiotics for the treatment of gut inflammation (20). Comparing to the non-challenged group, the *Megamonas* genus was significantly reduced in all challenged groups. *Megamonas* is a major propionate producer *via* methylmalonyl-CoA mutase, epimerase, and decarboxylase (22). Propionate is one of three (acetate, propionate, and butyrate) important short-chain fatty acids. SCFAs are essential for host metabolism, induce the differentiation of regulatory T cells to enhance the host health, and serve as energy and carbon sources for poultry (23–25).

*Bifidobacterium* genus was another beneficial taxa reduced in abundance in the *C. hepaticus* NSW44L challenged group. *Bifidobacterium* play a role in gut health promotion by increasing intestinal immunostimulation and producing host beneficial volatile fatty acids (26, 27). *Faecalibacterium* and *Bifidobacterium* are both anaerobic bacteria and have shown potential use as probiotics to improve human and animal health. Administration of strains of *B. breve* and *B. infantis* species showed body weight gain and prevented the deleterious effects and mortality due to *Salmonella* infection in chickens through competitive exclusion and the release of cytokines (28). Therefore, the reduction of SCFAs producer *Faecalibacterium, Bifidobacterium* and *Megamonas* in the SLD-challenged groups further demonstrates the negative effect of *C. hepaticus* on chickens. It will be interesting in future work to assess the SCFA levels in SLD affected birds.

*Ruminococcus torques* group and *Enorma* genera were also reduced in the NSW44L challenged group. A high abundance of these bacteria has previously been associated with better performance in poultry (29, 30). The role of *Enorma* in human and animal health has not been studied widely, however, Khan and Chousalkar have shown that the *Enorma* genus was also reduced from *Salmonella Typhimurium*-challenged group (31).

The small intestine is important for the digestion and absorption of nutrients, and histological alterations have been found to be related to changes in intestinal function (32). Longer intestinal villi increase the absorptive surface, resulting in increased absorption capacity of the intestine in chickens. Similarly, a higher villus height and crypt depth ratio are associated with a greater capacity for nutrient digestibility and absorption (33, 34). Although SLD-induction affected the gut microbiota of chickens, their small intestine structure was not significantly different in the villus height and ratio of villus height and crypt depth. This is in contrast to the effects induced by some pathogens that cause specific damage to the small intestine as part of the pathogenic processes that they initiate, for example, by *Clostridium perfringens* infections, where birds inoculated with 1 mL of 2.0 × 10^8^ cfu/mL showed decreased intestinal villus height and a lower ratio of villus height to crypt depth ratio (35).

In conclusion, *C. hepaticus* infection impacted the microbiota of chickens. They were most affected in the *C. hepaticus* NSW44L challenged group, which also had the highest lesion score amongst the challenged groups. The Shannon and Simpson diversity indexes were decreased in NSW44L challenged group, indicating lower bacterial diversity in infected birds. Some SCFAs producing bacteria were significantly reduced in all challenged groups. *C. hepaticus* might activate the host's innate immune responses, such as antimicrobial peptides, that could lead to the changes in the composition of the gut microbiota of the host. The change in caecal microbiota in an unhealthy direction may be one of the reasons for production loss during SLD outbreaks. Approaches to improve the birds' gut health during SLD outbreaks, such as through diet, probiotic supplementation, SCFA addition, and keeping the causes of stress to a minimum, may assist in the management of SLD.

## Data availability statement

The datasets presented in this study can be found in online repositories. The names of the repository/repositories and accession number(s) can be found at: https://www.ncbi.nlm.nih.gov/, PRJNA877767.

## Ethics statement

The animal study was reviewed and approved by the Wildlife and Small Institutions Animal Ethics Committee of the Victorian Department of Economic Development, Jobs, Transport and Resources (Approval number 14.16).

## Author contributions

TV and RM conceived the study. TV and LL performed the experiments. TW, AA, PS, TV, and RM carried out the chicken trial. TV and DS performed bioinformatics analysis of the sequencing data. TV and RP drafted the manuscript. RM and DS edited the manuscript. All authors have edited and approved the final manuscript.

## Conflict of interest

Authors AA, TW, and PS were employed by Scolexia Pty Ltd. The remaining authors declare that the research was conducted in the absence of any commercial or financial relationships that could be construed as a potential conflict of interest.

## Publisher's note

All claims expressed in this article are solely those of the authors and do not necessarily represent those of their affiliated organizations, or those of the publisher, the editors and the reviewers. Any product that may be evaluated in this article, or claim that may be made by its manufacturer, is not guaranteed or endorsed by the publisher.
